# Reaction‐Driven Self‐Organization of Nanocrystals by Kinetic Control of Inhomogeneous Photopolymerization

**DOI:** 10.1002/advs.78014

**Published:** 2026-09-30

**Authors:** Kazuto Uchida, Shiryu Kitajima, Koki Ito, Hideyuki Nakanishi

**Affiliations:** ^1^ Graduate School of Science and Technology Kyoto Institute of Technology Matsugasaki Kyoto Japan; ^2^ Faculty of Materials Science and Engineering Kyoto Institute of Technology Matsugasaki Kyoto Japan

**Keywords:** nanocrystals, patterning, perovskite quantum dots, photopolymerization, self‐assembly

## Abstract

Reaction‐driven self‐organization of nanocrystals offers a promising route to functional patterns, yet the kinetic principles governing this process remain poorly understood. Here, we investigate how CsPbBr_3_ perovskite quantum dots (PQDs) migrate and spontaneously organize under patterned‐light‐induced photopolymerization. Using a custom‐built digital micromirror device‐based light projection system combined with in situ confocal microscopy, we directly correlate the applied light field with nanocrystal transport. We show that PQD migration is driven by the time‐dependent generation of a monomer‐conversion difference between high‐ and low‐light‐intensity regions, but that the final pattern is not determined by this driving force alone. Under weak irradiation, redistribution of other mobile species relaxes the conversion difference, whereas under strong irradiation, rapid polymerization is inferred to reduce PQD mobility through viscosity buildup and solidification. Intermittent and periodic irradiation reveal that nanocrystal self‐organization is governed by a competition between buildup of the driving force and loss of mobility during solidification. These results establish a kinetic design principle for reaction‐driven nanocrystal patterning and show that photopolymerization can serve as a programmable reaction‐diffusion field for directing colloidal transport and self‐organization.

## Graphical abstract

Patterned light reorganizes initially uniform CsPbBr_3_ perovskite quantum dots into well‐defined structures during photopolymerization. Using a digital micromirror device, we show that this reaction‐driven self‐organization is governed by a competition between chemical‐potential‐driven migration and kinetic arrest caused by rapid solidification. Intermittent irradiation reveals how light programs colloidal transport.

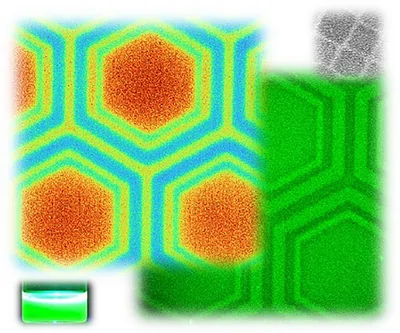

## 1 Introduction

The controlled spatial organization of nanocrystals is central to the development of functional nanoscale materials. Because nanocrystals exhibit size‐dependent optical, electronic, catalytic, and interfacial properties [1, 2, 3, 4], their collective performance depends not only on composition and size but also on how they are arranged in space. In particular, the ability to guide nanocrystals from an initially dispersed state into ordered or patterned structures is a key challenge in nanoscience and nanotechnology.

Photocurable resins provide an attractive platform for addressing this challenge. They enable rapid curing, are compatible with high‐throughput and energy‐efficient manufacturing, and, because of their solvent‐free nature, are also of interest as sustainable materials [5, 6, 7]. To expand their utility beyond conventional roles such as adhesion, coatings, and structural fabrication, however, additional functionalities must be integrated into these reactive media [8, 9, 10]. In this context, the incorporation and controlled organization of functional nanocrystals within photocurable resins represent a particularly promising direction.

Current strategies for nanocrystal organization are broadly divided into top‐down and bottom‐up approaches. Top‐down methods, including photolithography [11, 12], microcontact printing [13, 14], inkjet printing [15, 16], nanoimprint lithography [17, 18], and laser direct writing [19, 20], offer direct spatial definition and, in some cases, high resolution. However, these approaches often require multistep processing, specialized equipment, or carefully tuned inks and can be difficult to apply to fragile nanocrystals or solvent‐sensitive systems. Bottom‐up approaches based on self‐assembly [21, 22, 23, 24] or phase separation [25, 26, 27, 28] are attractive because they exploit spontaneous organization, but robust external control over the final structure remains challenging because the outcome emerges from coupled dynamic processes rather than direct writing.

In this context, nanocrystal organization driven by spatially modulated photopolymerization represents a distinctive and underexplored route [29, 30, 31, 32]. Under patterned irradiation, polymerization proceeds non‐uniformly, creating transient composition gradients in the evolving reaction medium. These gradients are generally considered to drive nanocrystal migration through differences in chemical potential, thereby transforming an initially homogeneous dispersion into a patterned structure. This process is particularly intriguing because it combines external programmability with spontaneous reaction‐driven transport: light encodes the spatial information, whereas the nanocrystals reorganize through transport within the reacting medium. In this sense, inhomogeneous photopolymerization offers a platform for reaction‐driven self‐organization of nanocrystals.

Previous studies from our group established the basic concept and key mechanistic features of photopolymerization‐driven nanocrystal organization. Reaction‐induced concentration differences were shown to drive nanocrystal migration, while rapid polymerization and solidification can kinetically restrict that migration [31, 32]. Subsequent digital micromirror device (DMD)‐based in situ observation of perovskite quantum dots (PQDs) further demonstrated that pattern formation reflects a competition between the thermodynamic promotion of migration and its kinetic limitation during polymerization [33]. However, these contributions were primarily inferred under continuous irradiation, where generation of the driving force and loss of particle mobility occur simultaneously. Their temporal interplay therefore remained difficult to disentangle experimentally. In particular, it remained unclear how PQD migration responds when ongoing photopolymerization is temporarily interrupted after a composition difference has been established, and how the timing of such perturbations influences the final organization.

Here, we investigate the kinetic principles governing reaction‐driven nanocrystal self‐organization during inhomogeneous photopolymerization. Using a custom‐built DMD‐based light projection system integrated with in situ confocal microscopy, we directly correlate the applied light field with the migration of CsPbBr_3_ PQDs. PQDs provide an excellent model system because their strong luminescence allows direct visualization of transport, while their practical relevance as process‐sensitive nanocrystals highlights the need for nonconventional patterning strategies. We show that nanocrystal organization is governed by a competition between the generation of a thermodynamic driving force and the kinetic loss of mobility during solidification. By introducing intermittent and periodic irradiation as controlled perturbations, we further establish a kinetic design principle for directing nanocrystal self‐organization in reactive media.

## 2 Results and Discussion

The photocurable solution, consisting of monomers, a photoinitiator, and CsPbBr_3_ PQDs, was introduced into a 5 µm gap between two coverslips separated by a spacer. The synthesized CsPbBr_3_ PQDs were characterized by TEM, optical spectroscopy, and x‐ray diffraction (Figure S1). The sample cell was mounted on the stage of an inverted confocal microscope, and patterned light with a peak wavelength of 454 nm was projected from above using a DMD‐based light‐projection system equipped with an LED light source; the DMD enables precise spatial control of light intensity [33, 34] and thereby systematic regulation of local polymerization kinetics. As an initial demonstration of this phenomenon, Figure 1 shows that PQDs initially dispersed homogeneously in the photocurable solution can be reorganized into a wide range of spatial patterns through DMD‐controlled inhomogeneous photopolymerization. In the following, the organization mechanism was investigated using a single representative pattern.

**FIGURE 1:**
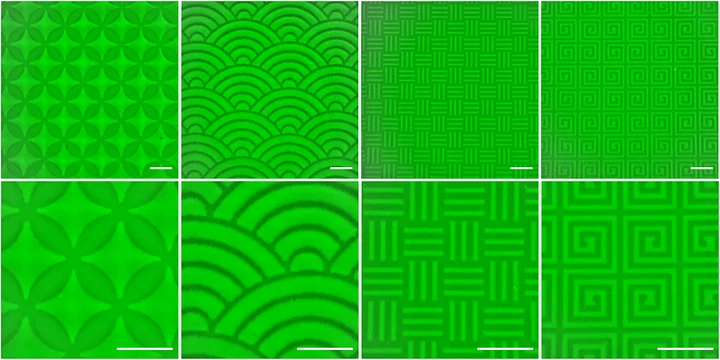
Representative examples of nanocrystal organization induced by DMD‐controlled inhomogeneous photopolymerization. The images in the bottom row show higher‐magnification views of the corresponding images in the top row. Scale bars are 400 µm.

Figure 2a–c show the light patterns applied to the photocurable solution, where the light intensity, *I*, was visualized in a separate experiment (Figure S2). Three different light patterns with overall low, medium, and high intensities, denoted as Pattern L, Pattern M, and Pattern H, respectively, were employed. Their 1D averaged intensity profiles, taken over the region of *y* = 175–275 µm, are shown in Figure 3a. In response to irradiation with these patterns, luminescent PQD patterns emerged, as shown in Figure 2d–f, where *I*_PQD_ represents the emission intensity of the PQDs. Importantly, the applied light pattern and the resulting PQD luminescence pattern could be analyzed in the same coordinate system, allowing direct correlation between the optical stimulus and the migration response.

**FIGURE 2:**
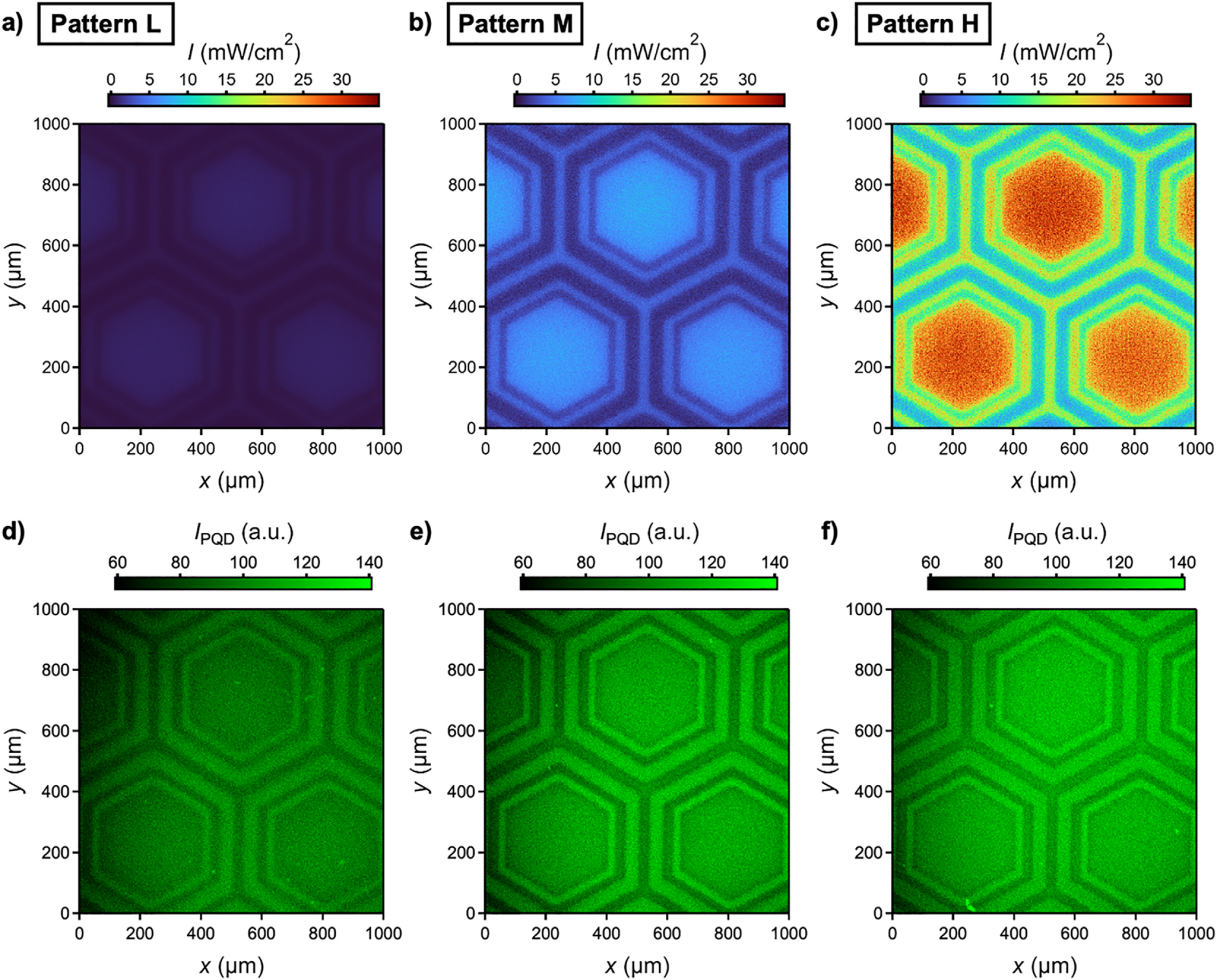
Comparison between the applied light patterns and the resulting PQD patterns. (a–c) Three different light patterns, denoted as Pattern L, Pattern M, and Pattern H, applied to the photocurable solution. These patterns have the same shape but differ in overall intensity. (d–f) PQD patterns obtained by irradiation with (a) Pattern L, (b) Pattern M, and (c) Pattern H, respectively.

**FIGURE 3:**
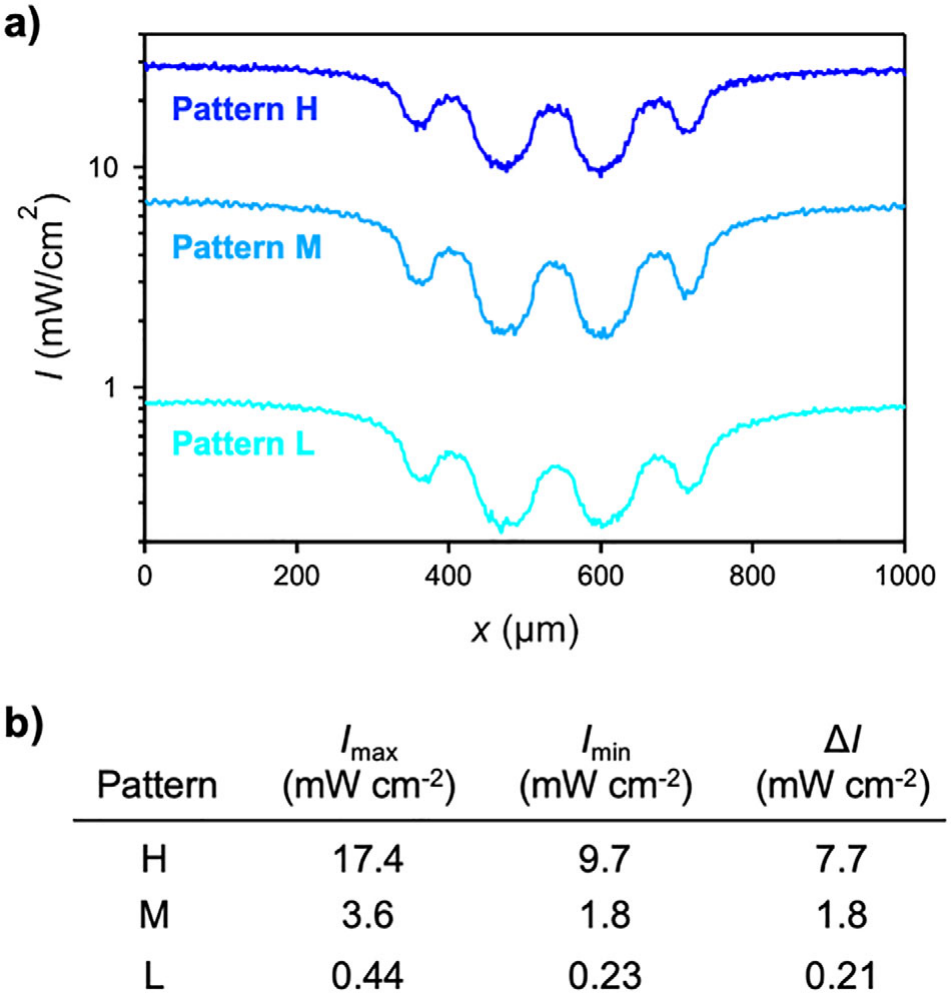
Details of the applied light patterns. (a) One‐dimensional averaged light intensity profiles of Patterns L, M, and H. (b) Maximum and minimum light intensities, *I*_max_ and *I*_min_, respectively, and their difference, Δ*I* (= *I*_max_−*I*_min_). The definitions of *I*_max_ and *I*_min_ are shown in Figure 4d.

The spatial resolution achieved in the present experiments is constrained by the projection optics, which broaden the light pattern delivered from the DMD to the sample. Because the projected light field itself is therefore not sufficiently fine to independently evaluate the intrinsic resolution of the material response, the respective contributions of DMD pixel size, optical diffraction, and transport within the reacting medium cannot be distinguished from the present data. Determining the ultimate resolution of this patterning process would require a dedicated high‐resolution projection system and systematic resolution measurements and is beyond the scope of the present study.

Figure 4a–c summarizes the correspondence between *I* and *I*_PQD_. The value of *I*_PQD_ is high in regions where *I* is low, indicating that the PQDs migrate from regions of high light intensity to those of low light intensity during photopolymerization. This migration direction is consistent with that reported for other photocurable systems containing different monomers, polymerization mechanisms (e.g., free‐radical or cationic polymerization), and nanocrystals, such as silver nanowires, gold nanoparticles, TiO_2_ nanoparticles, and alumina particles [31, 35]. Thus, the present result supports the general tendency that nanocrystals migrate toward low‐light‐intensity regions, that is, toward relatively monomer‐rich and less polymerized regions formed during irradiation.

**FIGURE 4:**
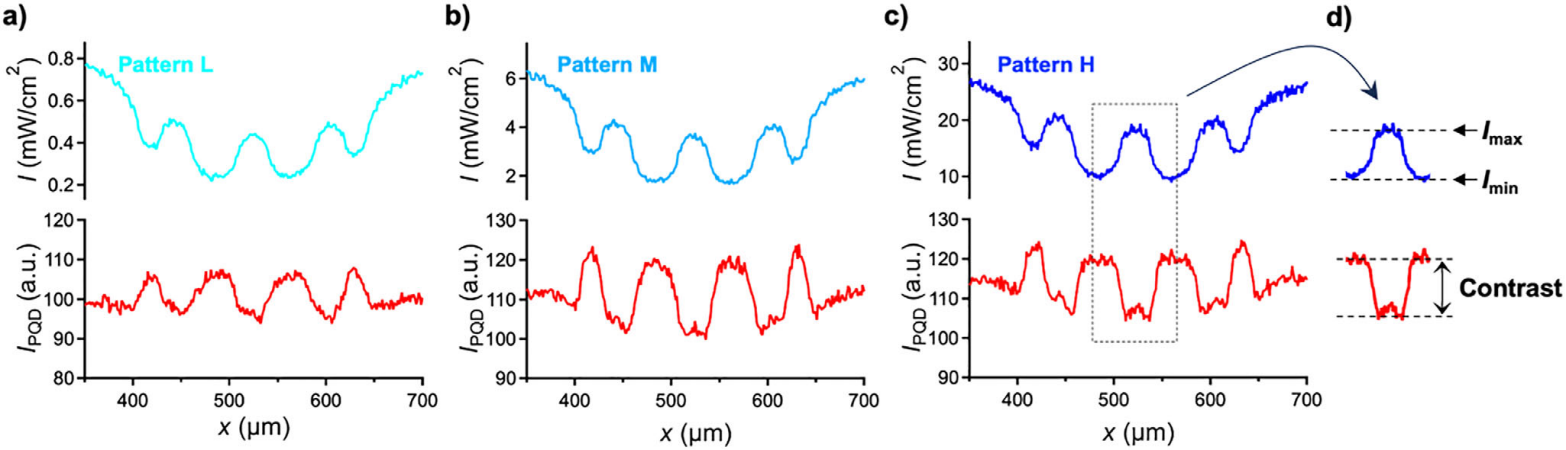
Comparison of the applied light patterns and the resulting PQD patterns using one‐dimensional averaged intensity profiles. (a–c) *I* and *I*_PQD_ for Patterns L, M, and H, respectively. (d) Schematic illustration showing the definitions of the maximum and minimum light intensities, *I*_max_ and *I*_min_, and the contrast of the PQD pattern.

This cross‐system invariance suggests that nanocrystal migration during photopolymerization involves a general physicochemical contribution rather than being governed solely by the specific chemistry of an individual photocurable system. The particles examined to date differ not only in composition and morphology but also in surface chemistry, including oleic acid/oleylamine‐capped CsPbBr_3_ PQDs, PVP‐coated silver nanowires, dodecanethiol‐capped gold nanoparticles, and TiO_2_ and Al_2_O_3_ particles [31, 35]. If system‐specific enthalpic interactions between the particle surface and the surrounding matrix were the dominant determinant of migration direction, stronger sensitivity to changes in surface and matrix chemistry, including possible directional reversals, might be expected. The absence of such reversals does not exclude enthalpic contributions, which may influence the magnitude and kinetics of migration. Rather, it supports a thermodynamic picture in which a general contribution associated with polymer‐chain formation—such as an entropic penalty imposed on dispersed species through excluded‐volume or size‐exclusion effects—provides a common bias toward polymer‐poor regions [36, 37, 38]. Accordingly, polymer‐rich regions generated during photopolymerization become thermodynamically less favorable for nanocrystals, driving their migration toward less‐polymerized regions. This interpretation is also consistent with previous studies on polymerization‐induced phase separation and photopolymerization, which have emphasized the thermodynamic role of network elasticity, thermodynamic compatibility, and the coupling of phase separation with polymerization kinetics, gelation, and mobility [39, 40, 41, 42, 43, 44].

To analyze the migration behavior quantitatively, the applied light pattern was characterized hereafter by the maximum and minimum light intensities, *I*_max_ and *I*_min_, respectively, whereas the degree of PQD migration was evaluated by the contrast of the PQD pattern, defined as the emission‐intensity difference in Figure 4d. The values of *I*_max_, *I*_min_, and the light‐intensity difference Δ*I* (= *I*_max_−*I*_min_) are summarized in Figure 3b.

Ideally, to elucidate the migration mechanism from a kinetic perspective, the time‐dependent composition difference between the high‐ and low‐light‐intensity regions, characterized by *I*_max_ and *I*_min_, should be correlated with the extent of PQD migration. However, it is experimentally difficult to determine the time‐dependent monomer conversion, *Φ*, separately for the *I*_max_ and *I*_min_ regions within the same photocurable solution. Therefore, *Φ* was measured independently at *I*_max_ and *I*_min_ in separate experiments, and the monomer‐conversion difference between the two regions, Δ*Φ*, was estimated, as shown in Figure 5a (Figure S3). This construction assumes that the local photopolymerization kinetics are governed primarily by the local irradiation intensity. However, it does not account for lateral redistribution of mobile components between regions with different irradiation intensities in the patterned sample. Therefore, the estimated Δ*Φ* should be regarded as an effective reference for the characteristic time scale of conversion‐difference development rather than as a direct measurement of the instantaneous conversion difference within the patterned sample.

**FIGURE 5:**
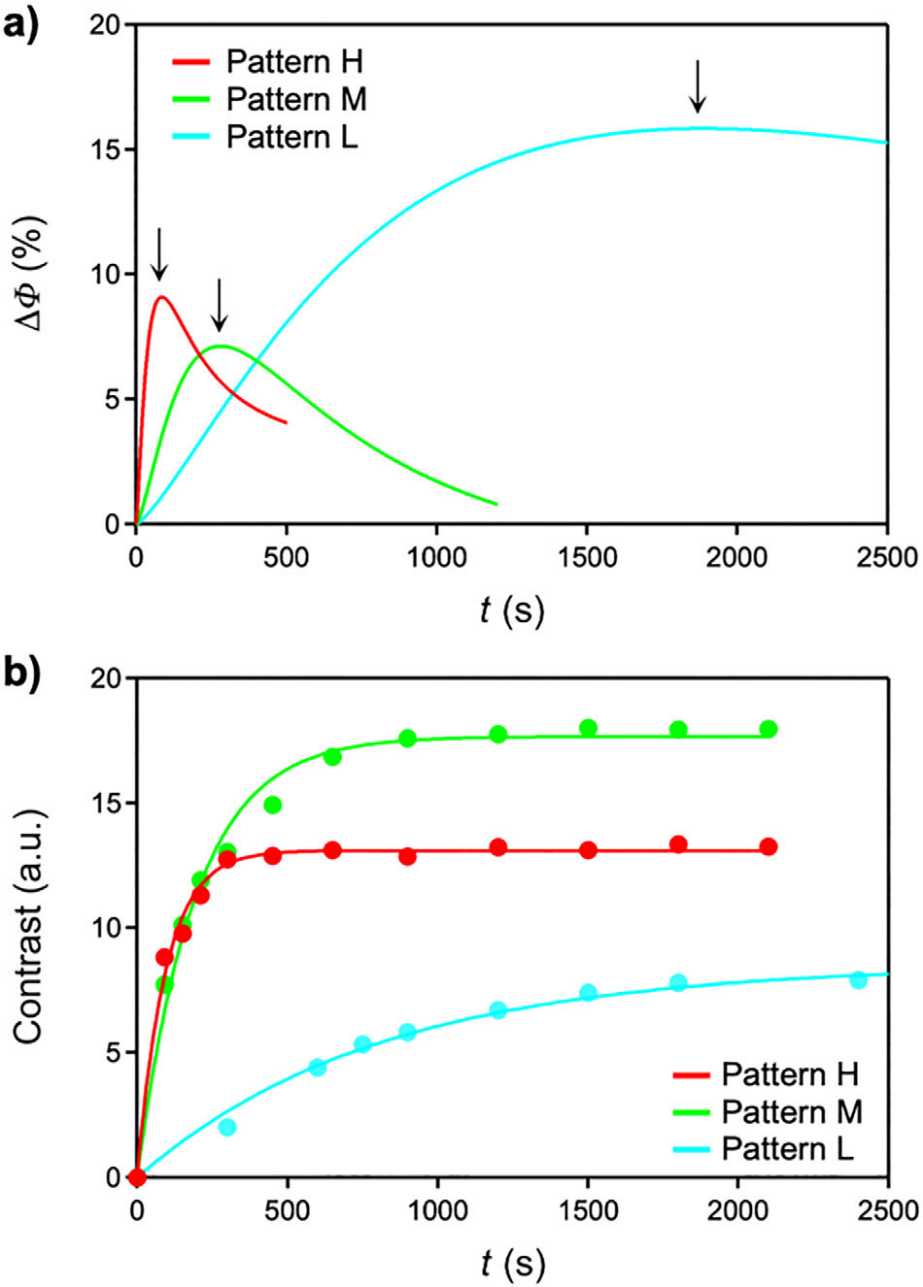
Correlation between polymerization kinetics and PQD migration. Time dependences of (a) the monomer‐conversion difference, Δ*Φ*, and (b) the contrast of the PQD pattern. The solid lines in (b) represent the curves obtained by curve fitting.

The time at which Δ*Φ* reaches its maximum, indicated by the arrows in Figure 5a and defined as *t*_Δ_ *_Φ_*_, max_, was 1865, 280, and 85s for Patterns L, M, and H, respectively. Thus, *t*_Δ_ *_Φ_*_, max_ decreases in the order Pattern L > Pattern M > Pattern H, indicating that the conversion difference between the *I*_max_ and *I*_min_ regions develops more rapidly as the overall light intensity increases. Notably, the early‐stage temporal profiles of Δ*Φ* differ markedly among the three patterns: Δ*Φ* rises sharply for Pattern H, more gradually for Pattern M, and most slowly for Pattern L, reaching its maximum much later.

Figure 5b shows the time evolution of the PQD contrast, which represents the extent of PQD migration. Comparison of Figure 5a,b reveals that PQD migration terminates earlier as *t*_Δ_ *_Φ_*_, max_ becomes shorter, that is, in the order Pattern L, Pattern M, and Pattern H. This result indicates that the kinetics of PQD migration are closely coupled to the rate at which the conversion difference is generated between the two regions. In other words, the migration process becomes faster when the compositional asymmetry induced by photopolymerization develops more rapidly.

To further quantify this relationship, the migration kinetics were characterized by the time constant, *τ*_c_, obtained by fitting the time‐dependent contrast data to an exponential function, *f*(*t*) = *C*[1‐exp(‐*t*/*τ*_c_)], where *C* is the maximum contrast ultimately achieved. As shown in Figure 6, *τ*_c_ exhibits a clear linear relationship with *t*_Δ_ *_Φ_*_, max_. This result strongly supports the conclusion that PQD migration is governed by the time‐dependent generation of the conversion difference and, hence, by the spatially inhomogeneous composition produced during photopolymerization, which acts as the driving force for migration.

**FIGURE 6:**
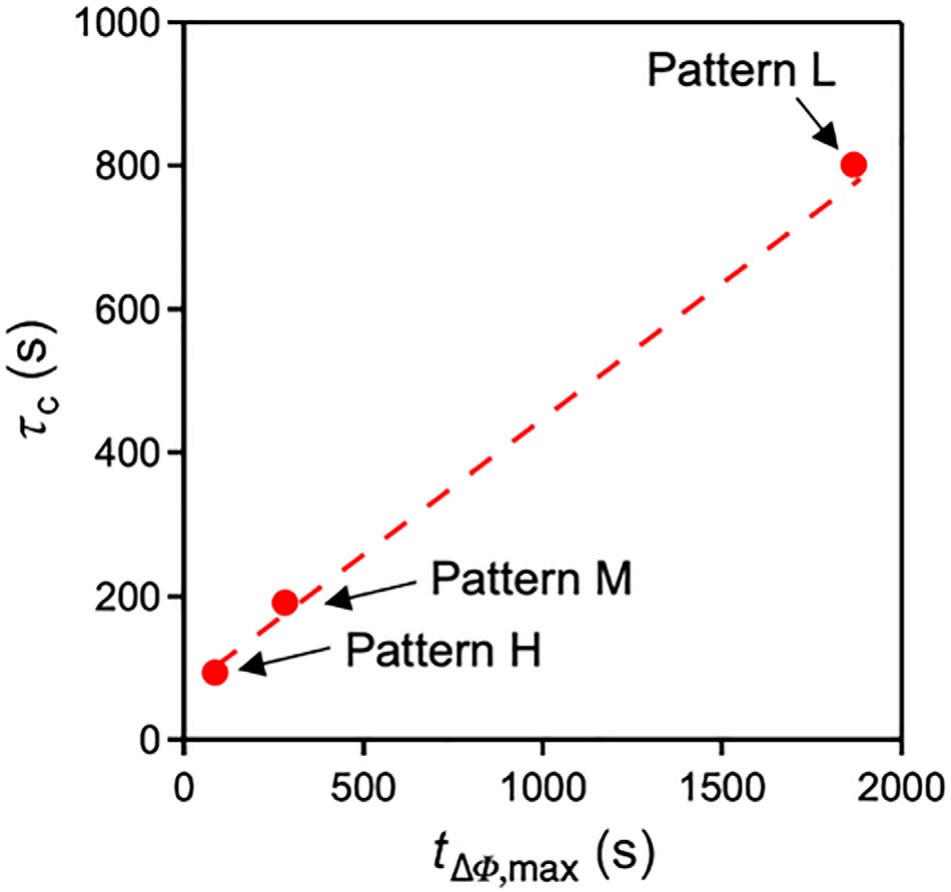
Relationship between *τ*_c_ and *t*_Δ_ *_Φ_*_, max_, showing the correlation between the kinetics of PQD migration and those of the monomer‐conversion difference.

Intuitively, the extent of PQD migration would be expected to increase with increasing compositional inhomogeneity, as reflected in the conversion difference Δ*Φ*. However, this simple expectation does not always hold. As shown in Figure 5a, Pattern L produces the largest conversion difference, Δ*Φ* = 16% (*t*_Δ_ *_Φ_*_, max_ = 1865s), among the three light patterns, yet it gives the smallest PQD contrast (*C* = 8.6) in Figure 5b. This apparent discrepancy indicates that the magnitude of Δ*Φ* alone does not determine PQD migration. Although Pattern L eventually gives the largest estimated Δ*Φ*, the slow development of this conversion difference allows substantial diffusive relaxation of other mobile species during the period in which PQDs remain mobile. Therefore, the effective driving force experienced by PQDs cannot be represented solely by the maximum value of Δ*Φ*, but must depend on how the conversion difference is generated, maintained, and relaxed during polymerization.

In principle, mobile components of the reacting mixture, including monomers, oligomeric species, and photoinitiator‐derived species, may also redistribute under patterned irradiation. In particular, the overall light intensity of Pattern L is the lowest among the three patterns (cf. Figure 3a), and polymerization therefore proceeds slowly, so that the viscosity of the solution remains low and the components retain high mobility. Under such conditions, redistribution of these mobile species may attenuate the development of the conversion difference and thereby suppress the extent of PQD migration. To examine whether species other than the PQDs can redistribute under patterned irradiation, we prepared another photocurable solution in which fluorescent monomers, shown in Figure 7a, were homogeneously dissolved in place of the PQDs and irradiated the solution with patterned light. If no redistribution of the monomers occurred, the fluorescence intensity would remain spatially uniform throughout the resulting film. Instead, a fluorescent pattern emerged upon film formation (Figure 7b). This experiment specifically demonstrates redistribution of the labeled monomer but does not resolve the individual contributions of monomer, oligomer/polymer, and photoinitiator transport to relaxation of the conversion difference. We therefore use this result as evidence that redistribution is not limited to the PQDs and that multicomponent transport may contribute to relaxation of the conversion difference, rather than as a quantitative decomposition of the individual transport processes. Thus, polymerization kinetics also play an important role in effectively developing and maintaining the conversion difference and, in turn, determining the extent of PQD migration.

**FIGURE 7:**
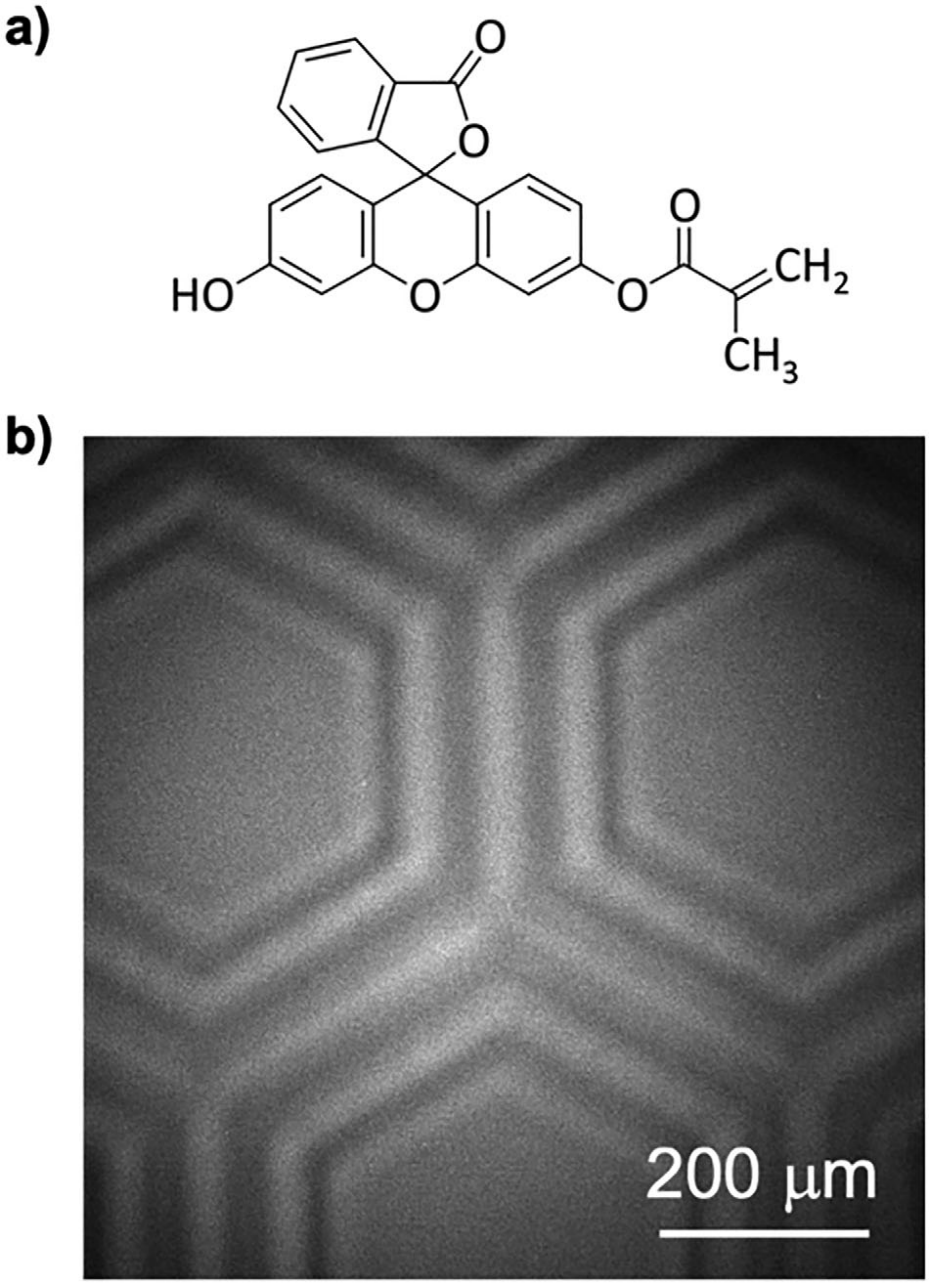
Diffusion of chemical species under patterned irradiation. (a) Chemical structure of the fluorescent monomer used to evaluate diffusion behavior. (b) Fluorescence pattern formed under patterned irradiation.

The contrast of the PQD pattern increases as the overall light intensity is raised from Pattern L to Pattern M (cf. Figure 5b), presumably because the conversion difference develops more effectively. In contrast, a further increase in light intensity from Pattern M to Pattern H reduces the contrast (cf. Figure 5b). For Pattern H, polymerization proceeds so rapidly that the solution is quickly transformed from a liquid into a solid. As a result, the mobility of the PQDs rapidly decreases, and their migration is likely to be kinetically hindered, leading to lower contrast.

To gain further insight into this kinetic effect, we temporarily interrupted the patterned irradiation to suspend polymerization, kept the solution in the dark while monitoring PQD migration, and then resumed the patterned irradiation to complete film formation. Figure 8 shows the results of these intermittent‐irradiation experiments, in which the dark period was fixed at 120s, whereas the time at which light irradiation was stopped was varied to correspond to the late, middle, and early stages of polymerization: (a) 210s, (b) 80s, and (c) 40s, respectively. The shaded regions in Figure 8 represent the dark periods, during which the contrast continues to increase, indicating that PQD migration proceeds even in the absence of light. These observations support an interpretation in which viscosity buildup and eventual solidification during polymerization progressively reduce PQD mobility, whereas the conversion difference provides the driving force for pattern formation. Because local viscosity and PQD diffusivity were not measured directly, this mobility‐loss mechanism should be regarded as an experimentally supported inference from the intensity‐dependent and intermittent‐irradiation responses rather than as a directly quantified relationship. Thus, inhomogeneous photopolymerization plays a dual role: it generates the thermodynamic driving force for PQD migration while simultaneously imposing an inferred kinetic constraint associated with the progression of polymerization.

**FIGURE 8:**
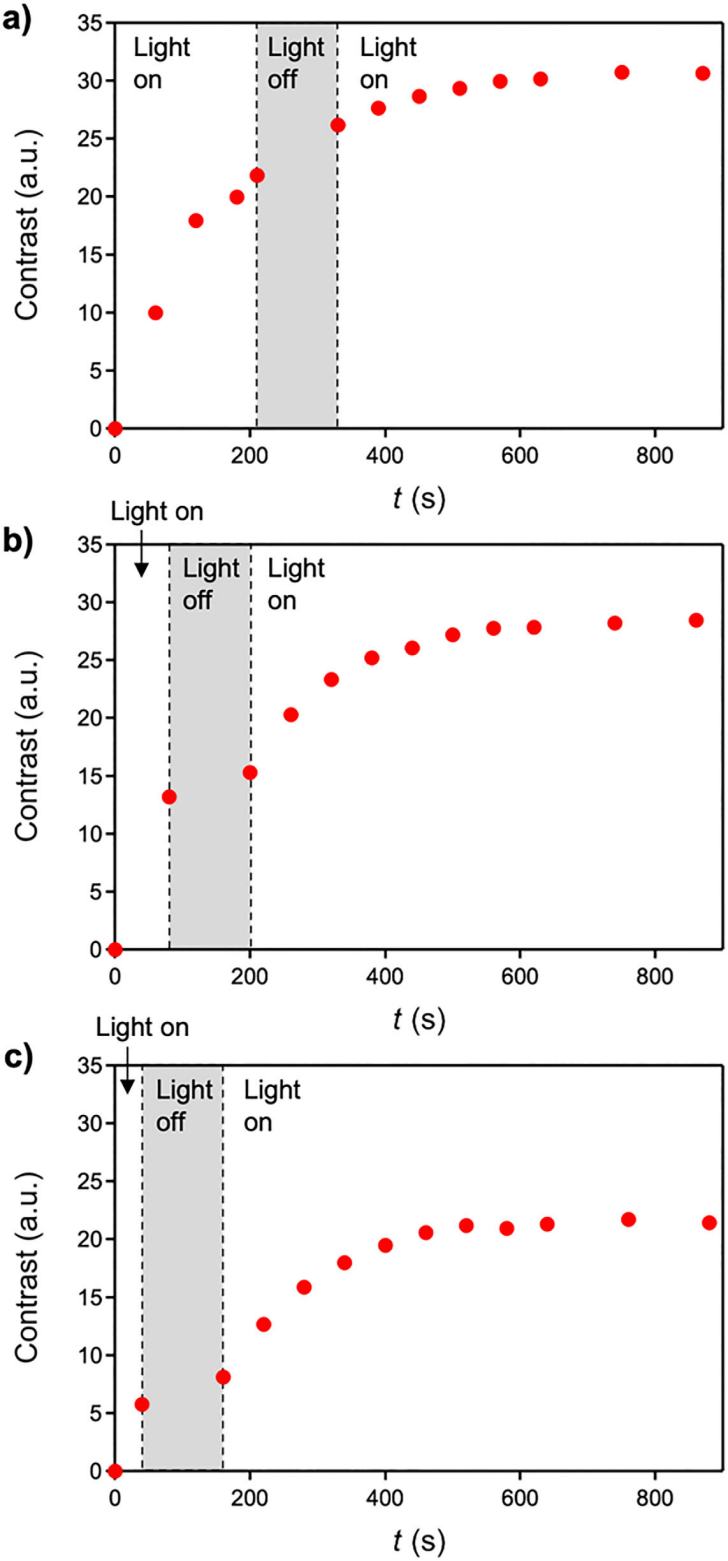
Time evolution of PQD contrast under intermittent patterned irradiation. Patterned irradiation was temporarily interrupted for 120s and then resumed. The light‐off timing was varied so as to correspond to different stages of polymerization: (a) 210s, (b) 80s, and (c) 40s after the start of irradiation. The shaded regions indicate the dark periods during which the patterned light was turned off.

Circumventing the kinetic constraint while maintaining the thermodynamic driving force for PQD migration is the key to enhancing pattern contrast. From this perspective, intermittent irradiation can be an effective means of generating high‐contrast patterns. Figure 9 summarizes the final contrast obtained in the intermittent‐irradiation experiments shown in Figure 8; the change in contrast is expressed as a percentage relative to that obtained under continuous irradiation, which was used as the reference.

**FIGURE 9:**
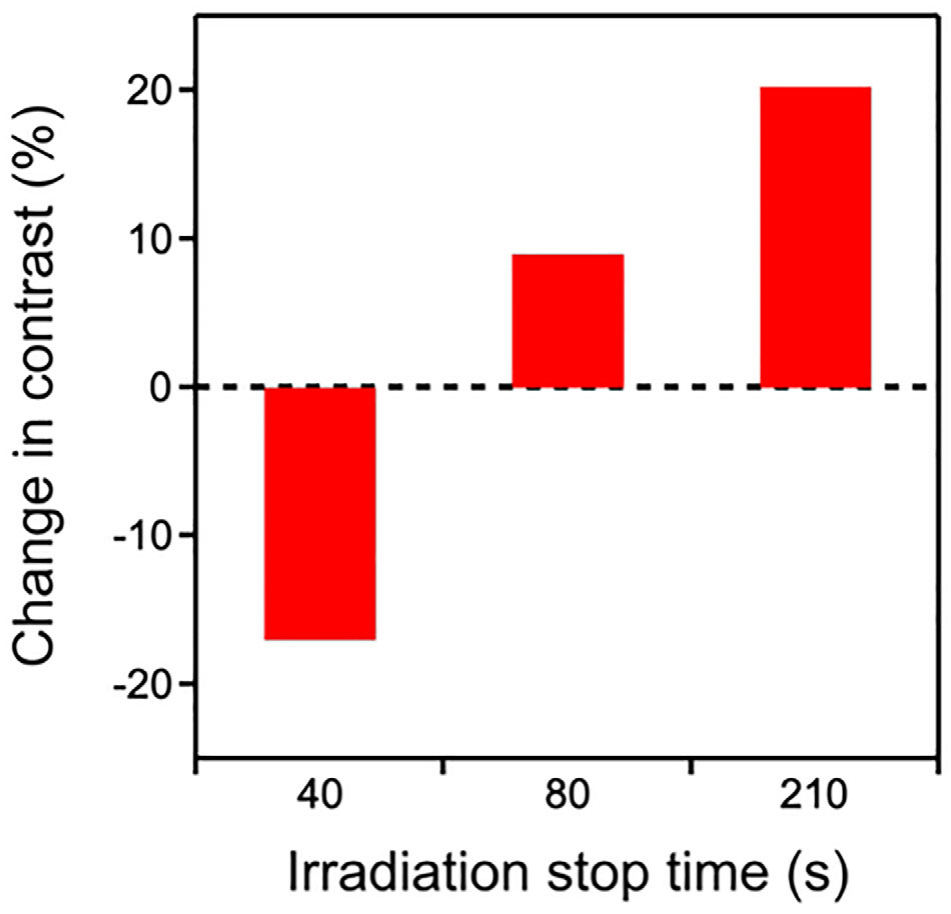
Relative change in the final PQD‐pattern contrast induced by intermittent irradiation. The values are given with respect to the contrast obtained under continuous irradiation.

Contrary to expectation, the final contrast becomes even lower (−17%) when irradiation is interrupted at 40s, that is, at the early stage of polymerization. Although the contrast increases slightly during the dark period (cf. Figure 8c), the final contrast remains lower than that obtained under continuous irradiation. This observation indicates that the contrast does not effectively increase during the second irradiation following the intermission, implying that the conversion difference is not efficiently re‐established in the second irradiation step. A plausible explanation is that, during the light‐off period, chemical species involved in polymerization diffuse in a manner that diminishes the conversion difference generated during the initial irradiation. Such behavior is reasonable because, at the early stage of polymerization, all components still retain high mobility.

By contrast, when irradiation is interrupted at 210s, that is, at the late stage of polymerization, the contrast increases markedly during the dark period (cf. Figure 8a), and the final contrast is improved by 20% relative to that obtained under continuous irradiation (cf. Figure 9). These results suggest that the effect of intermittent irradiation is governed by a competition between two processes during the dark period: continued PQD migration driven by the conversion difference established during the initial irradiation, and relaxation of that conversion difference through the diffusion of other chemical species. When irradiation is interrupted too early, the conversion difference is still insufficiently developed and is readily dissipated during the dark period, so that the driving force for PQD migration is lost. In contrast, when irradiation is interrupted at a later stage, the conversion difference has already developed sufficiently, while the temporary cessation of polymerization suppresses the viscosity increase that would otherwise kinetically hinder PQD migration. As a result, PQD migration can proceed efficiently in the dark, leading to enhanced final contrast.

On the basis of this interpretation, we next examined a periodic ON/OFF irradiation mode, in which the light‐on and light‐off processes were repeated with a defined period (Figure 10a). As shown in Figure 10b, the final contrast exhibits a maximum at a short period and then decreases monotonically with increasing period. This behavior can also be understood in terms of the same competition operating during each light‐off interval. At short periods, the light‐off time is long enough to relax the kinetic constraint imposed by the viscosity increase, but still short enough to prevent substantial dissipation of the conversion difference. Under these conditions, PQD migration is effectively promoted, resulting in enhanced contrast. In contrast, as the period becomes longer, the conversion difference generated during each light‐on interval is increasingly diminished by the diffusion of other chemical species during the subsequent light‐off interval. Consequently, the thermodynamic driving force for PQD migration is progressively lost, and the final contrast decreases. The observed maximum at a short period therefore indicates the existence of an optimal temporal balance between relaxation of the kinetic constraint and preservation of the driving force for migration. The optimum period of 10 s identified under the present conditions should not be regarded as universal. Rather, it is expected to depend on parameters that determine the characteristic timescales for conversion‐difference generation, compositional relaxation, and loss of PQD mobility, including the overall light intensity, monomer/crosslinker concentration and composition, PQD size, and sample geometry. A systematic determination of these dependencies lies beyond the scope of the present study.

**FIGURE 10:**
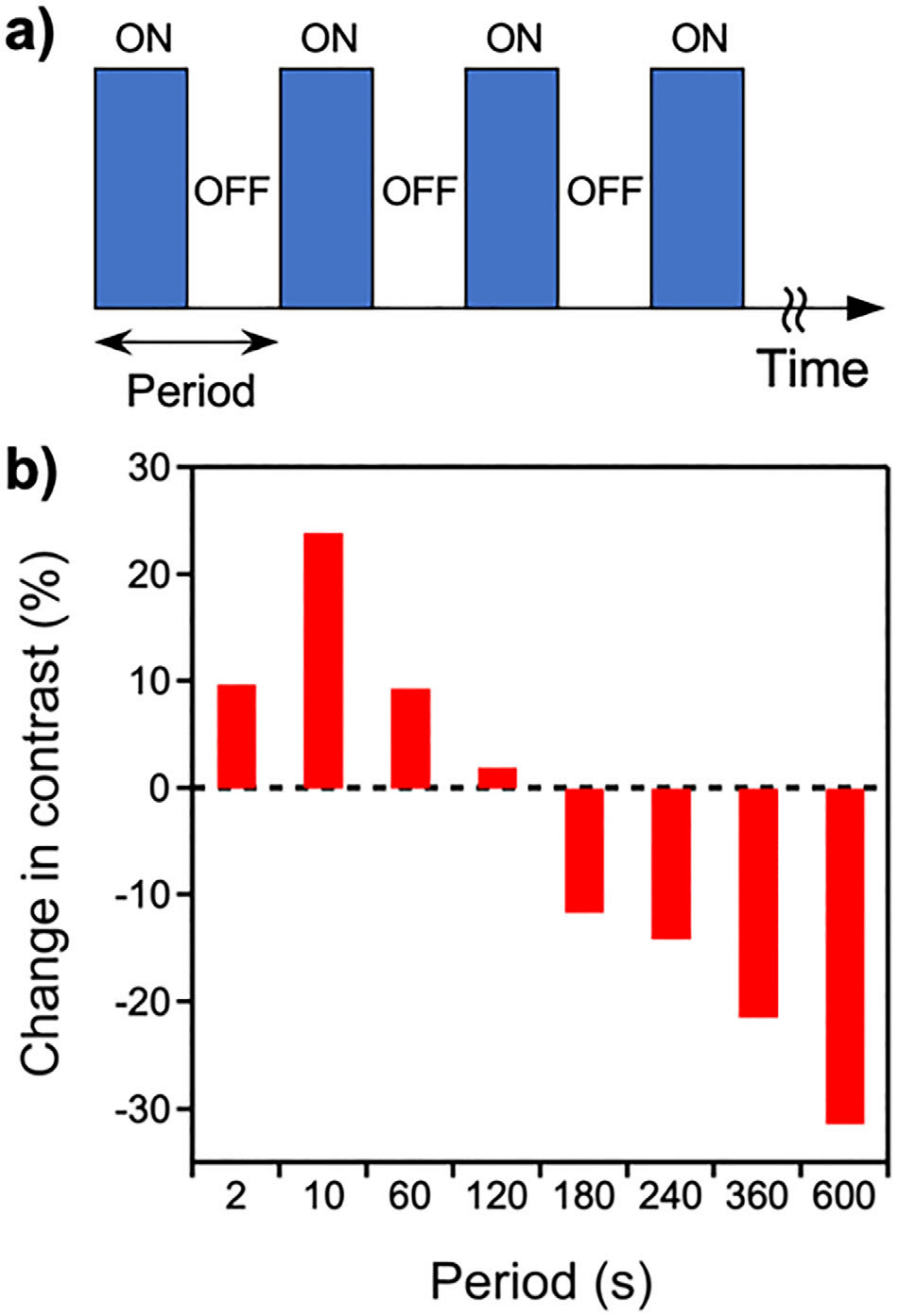
Dependence of the final PQD‐pattern contrast on the period of periodic ON/OFF irradiation. The light‐on and light‐off processes were repeated periodically during patterned irradiation. (a) Schematic illustration of the periodic irradiation. (b) Relative change in the final PQD‐pattern contrast as a function of the irradiation period.

Thus, the present work extends our previous identification of thermodynamic and kinetic contributions by directly interrogating their temporal interplay. Intermittent and periodic irradiation provide controlled temporal perturbations that reveal how continued PQD migration competes with relaxation of the driving force and progressive mobility loss, thereby establishing a temporal strategy for controlling nanocrystal organization.

More importantly, the present system involves tightly coupled polymerization, multicomponent mass transport, viscosity growth, and solidification. A quantitatively predictive treatment would require the independent spatiotemporal determination or validation of local conversion, viscosity, PQD mobility, and the concentrations and transport properties of multiple mobile reactive components. The fluorescent‐monomer experiment demonstrates that at least one reactive component redistributes under patterned irradiation; moreover, because the local concentrations of the photoinitiator and crosslinker influence polymerization kinetics, their possible redistribution would also have to be considered. These quantities cannot presently be constrained independently with sufficient spatial and temporal resolution within the same patterned sample. Consequently, the available PQD‐contrast data and the effective conversion difference estimated from separate homogeneous‐irradiation experiments cannot uniquely identify a theoretical model: different model structures or parameter sets could reproduce the same observations, making such fitting descriptive rather than predictive. The perturbation‐based experimental approach adopted here therefore provides a practical means of identifying the kinetic contribution without introducing an underdetermined model. Accordingly, the present work provides an experimentally supported kinetic interpretation rather than a fully predictive theoretical description.

## 3 Conclusion

In conclusion, we have elucidated the kinetic factors governing the reaction‐driven self‐organization of CsPbBr_3_ perovskite quantum dots during inhomogeneous photopolymerization. By combining a custom‐built DMD‐based light‐projection system with in situ confocal microscopy, we directly correlated the applied light field with the migration behavior of PQDs and clarified how polymerization kinetics regulate the emergence of luminescent nanocrystal patterns.

The results reveal that PQD migration is closely coupled to the time‐dependent generation of the monomer‐conversion difference between high‐ and low‐light‐intensity regions. At the same time, the migration behavior cannot be understood solely in terms of this thermodynamic driving force. The increase in viscosity accompanying polymerization is inferred to impose a kinetic constraint on PQD migration, whereas redistribution of other mobile species can attenuate the conversion difference itself. Thus, inhomogeneous photopolymerization plays a dual role: it generates the driving force for nanocrystal organization while simultaneously limiting nanocrystal transport through kinetic arrest.

On the basis of this mechanistic understanding, we demonstrated that intermittent and periodic ON/OFF irradiation provide effective means of enhancing pattern contrast by optimizing the temporal balance between preservation of the driving force and relaxation of the kinetic constraint. More importantly, the present study shows that even in a system for which a fully quantitative spatiotemporal description is practically unattainable, the essential role of kinetics can still be uncovered experimentally by introducing well‐designed irradiation perturbations. The present findings therefore not only deepen the understanding of nanocrystal organization in reactive soft materials but also establish a practical experimental framework for analyzing similarly complex photopolymerization‐driven transport phenomena. More broadly, this study identifies photopolymerization as a programmable reaction‐diffusion field for directing colloidal transport and nanocrystal self‐organization. This framework should facilitate the design of advanced organization and patterning strategies for functional nanocrystals, particularly process‐sensitive materials such as perovskite quantum dots that are difficult to structure using conventional lithographic or printing approaches.

## Author Contributions

**Kazuto Uchida**: Writing – review and editing, data curation, investigation, validation, visualization. **Hideyuki Nakanishi**: writing – review and editing, validation, conceptualization, methodology, supervision, funding acquisition, project administration, writing – original draft. **Shiryu Kitajima**: writing – review and editing, data curation, investigation, validation, visualization. **Koki Ito**: writing – review and editing, data curation, investigation, validation, visualization.

## Conflicts of Interest

The authors declare no conflicts of interest.

## Supporting information




**Supporting File**: advs78014‐sup‐0001‐SuppMat.pdf.

## Data Availability

The data that support the findings of this study are available from the corresponding author upon reasonable request.
