# Supplementary material for: Reaction‐Driven Self‐Organization of Nanocrystals by Kinetic Control of Inhomogeneous Photopolymerization
Source: Adv Sci (Weinh). 2026 Sep 30:e78014. Online ahead of print. doi: 10.1002/advs.78014 (PMC13627771; doi:10.1002/advs.78014)
Supplement: Supplementary file 1 [file ADVS-9999-e78014-s001.pdf]

Supporting Information

Reaction-Driven Self-Organization of Nanocrystals by Kinetic Control of Inhomogeneous Photopolymerization

Kazuto Uchida,[1] Shiryu Kitajima,[1] Koki Ito,[1] and Hideyuki Nakanishi*[1,2]

[1] Graduate School of Science and Technology, Kyoto Institute of Technology, Matsugasaki, Kyoto 606-8585 (Japan)

[2] Faculty of Materials Science and Engineering, Kyoto Institute of Technology, Matsugasaki, Kyoto 606-8585 (Japan)

* Correspondence to: hnakanis@kit.ac.jp

Synthesis of $CsPbBr_3$

$CsPbBr_3$ was prepared using a modified procedure based on a previously reported method.[1] Cs_2CO_3 (0.407 g, TCI), 1-octadecene (ODE; 20 mL, Sigma-Aldrich), and oleic acid (OA; 1.25 mL, Sigma-Aldrich) were added to a three-neck round-bottom flask. The flask was then placed in an oil bath set at 120 °C, and the mixture was degassed under vacuum for 1 h. Afterward, the temperature was raised to 150 °C under a N_2 atmosphere to generate Cs oleate.

Separately, $PbBr_2$ (0.138 g, Sigma-Aldrich) was combined with ODE (10 mL) in another flask. This mixture was degassed under vacuum at 120 °C for 1 h, followed by the addition of OA (1.0 mL) and oleylamine (1.0 mL) under N_2. Once $PbBr_2$ had fully dissolved, the temperature was increased to 160 °C, and the Cs-oleate solution (0.8 mL) was rapidly injected. After 5 s, the reaction flask was immediately cooled in an ice–water bath to stop the reaction. The resulting mixture was centrifuged at 4000 rpm for 5 min to collect $CsPbBr_3$ precipitates. The supernatant was removed, and the obtained $CsPbBr_3$ was uniformly redispersed in hexane (Figure S1a).

The size of $CsPbBr_3$ was estimated to be approximately 10 nm based on transmission electron microscopy observations (TEM; JEOL JEM-2100; Figure S1b). The absorption and emission spectra of $CsPbBr_3$ dispersed in hexane were measured using a Shimadzu UV-2700 spectrophotometer and a Shimadzu RF-5300PC spectrofluorophotometer, respectively (Figure S1c). The absorption and emission wavelengths were nearly identical to those previously reported. Furthermore, the formation of $CsPbBr_3$ was verified by X-ray diffraction analysis performed with a Rigaku MiniFlex 600 X-ray diffractometer using Cu Kα radiation (Figure S1d).

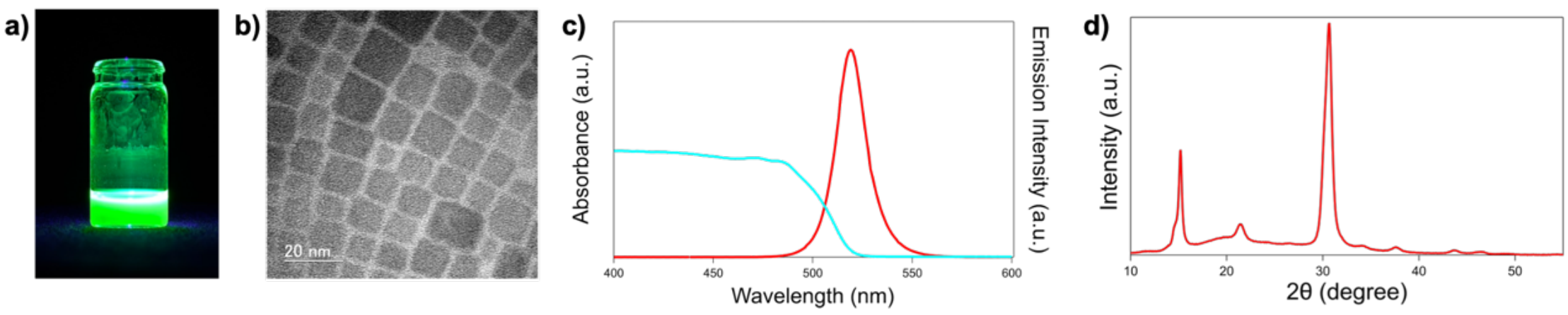

Figure S1. (a) Photograph of $CsPbBr_3$ dispersion in hexane under UV illumination. (b) TEM image of $CsPbBr_3$. (c) Absorbance spectrum (blue curve) and emission spectrum (red curve) of $CsPbBr_3$. (d) XRD pattern of $CsPbBr_3$.

Preparation of the photocurable solution

A $CsPbBr_3$ dispersion in hexane was homogeneously mixed with a liquid methacrylate monomer (UC-102M, Kuraray), after which hexane was removed under vacuum. Phenylbis(2,4,6-trimethylbenzoyl)phosphine oxide (TCI), used as a photoinitiator, and nonanediol diacrylate (TCI), used as a crosslinker, were then uniformly dissolved in the resulting $CsPbBr_3$ dispersion in the monomer. Thus, a photocurable solution was prepared with a weight ratio of monomer/crosslinker/$CsPbBr_3$/photoinitiator = $3.0/3.0/1.0/3.0 \times 10^{-2}$.

Visualization of the light intensity

The light intensity, $I(x,y)$, was determined using a photosensitive film based on a modified procedure reported in the literature.[2] Briefly, $I(x,y)$ was back-calculated from the increase in the fluorescence intensity of the photosensitive film. Rhodamine B (0.014 g, Fujifilm Wako Pure Chemical) was dissolved in 126 mL of methanol. Then, 1.0 g of the Rhodamine B solution was homogeneously mixed with 1.26 mL of methanol containing 1.1g of polyvinylpyrrolidone (MW 55,000, Sigma-Aldrich) and 0.055g of a photoacid generator (CPI-410S, SAN-APRO). Next, 30 μL of the polymer solution was drop-cast onto a coverslip, and the residual methanol was evaporated under vacuum. The prepared film was molded into a uniform-thickness film using a spacer by pressing it between glass slides at 160 ºC. Thus, a photosensitive film consisting of polyvinylpyrrolidone containing the photoacid generator and Rhodamine B was prepared.

The photosensitive film was irradiated with uniform light of a given intensity, without a patterned light field, using the DMD-based light-projection system, and the increase in the fluorescence intensity of Rhodamine B relative to its initial intensity, I_f, was monitored as a function of irradiation time. The increase in fluorescence intensity was presumably due to a structural change of Rhodamine B induced by acid generation under irradiation.[3] Analogous experiments were performed at different irradiation intensities, expressed in mW/cm^2, which were converted to the total energy dose, E, in mJ/cm^2 based on the irradiation time. I_f increased exponentially with E, collapsing onto a single curve irrespective of the irradiation intensity, and was fitted with the following exponential function: $I_f = (1+A_f)-A_f\exp(-E/\tau)$, where A_f is the maximum increase in I_f achieved at $E=\infty$, and τ is a constant, as shown by the solid curve in Figure S2.

The photosensitive film was then irradiated with the patterned light fields, Pattern L, Pattern M, and Pattern H, and I_f was obtained for each pattern. Finally, I_f was converted to the light intensity at each pixel based on the irradiation time using the universal calibration curve shown in Figure S2.

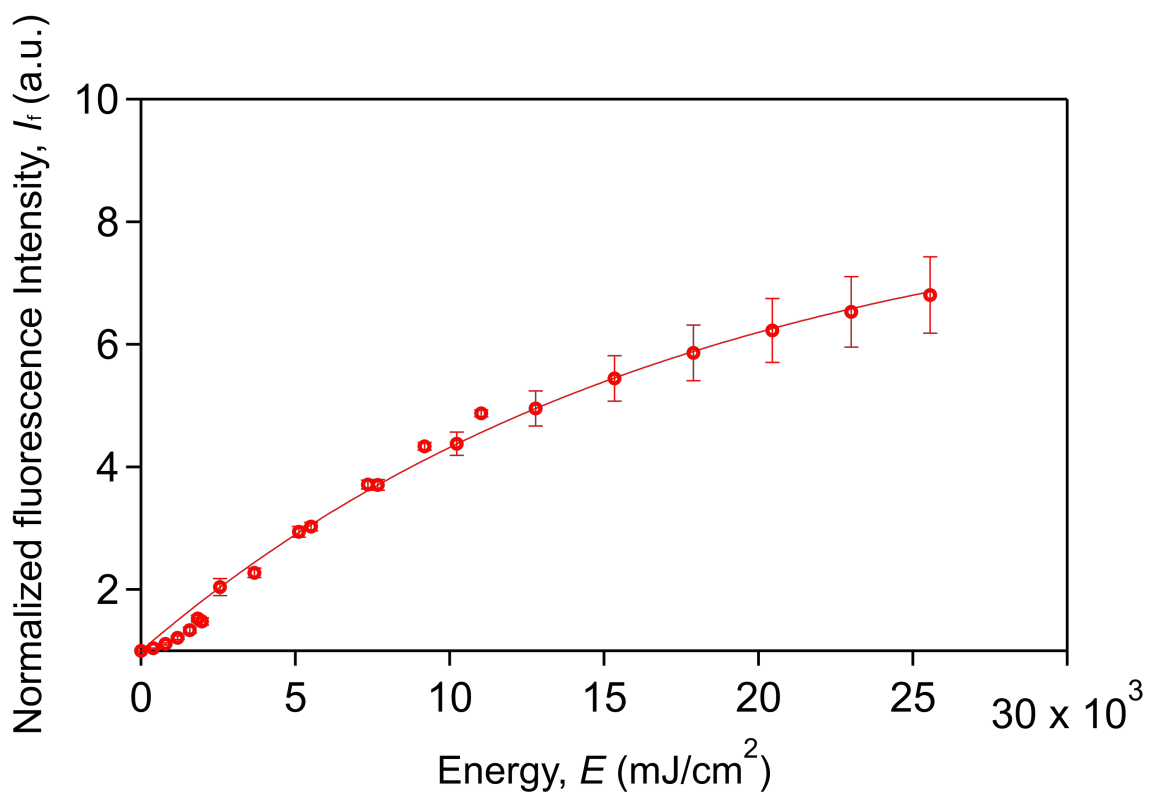

Figure S2. Normalized fluorescence intensity I_f as a function of the total energy E.

Determination of the time-dependent monomer-conversion difference

The monomer conversion, Φ, was determined using a Fourier transform infrared spectrometer (FT/IR-6600, JASCO). The photocurable solution was sealed in a 5µm-thick gap between two KBr plates (2000-0047, JASCO). The prepared sample cell was mounted in a holder (2000-0101, JASCO) and placed under illumination to promote polymerization. The photocurable solution was irradiated with uniform light of a given intensity, which was adjusted to I_{max} or I_{min} corresponding to each light pattern. The carbon double bonds in the monomer and crosslinker disappear during polymerization. Thus, Φ was evaluated by measuring the decrease in C=C absorption according to the following equation: $\Phi = [(A_0\text{-}A_t)/A_0]\times 100$, where A_0 and A_t are the C=C absorption intensities near 1630 cm^{-1} before irradiation and during irradiation, respectively. A_0 and A_t were obtained after normalization to the C=O absorption near 1720 cm^{-1}, because the C=O groups remain intact during polymerization and are therefore suitable as an internal reference.

Figure S3a-c show the monomer conversion, Φ, as a function of irradiation time, t, for Patterns L, M, and H, respectively. Φ was obtained under irradiation at light intensities corresponding to I_{max} and I_{min} for each pattern (cf. Figure 2b). The solid curves in Figure S3a-c were obtained by fitting the data to the following equation: $f(t) = \Phi_{max}[t^n/(t^n+K^n)]$, where Φ_{max} is the monomer conversion at $t=\infty$, and K and n are fitting parameters. The $\Delta\Phi$ curves shown in Figure 5a were obtained from the difference between the two fitted curves shown in each panel.

The difference between the fitted conversion curves provides an effective estimate of the conversion asymmetry expected from the local irradiation intensities I_{max} and I_{min}. This approximation assumes that the local conversion kinetics are governed primarily by the local irradiation intensity and does not account for lateral redistribution of mobile components between neighboring regions in the patterned sample.

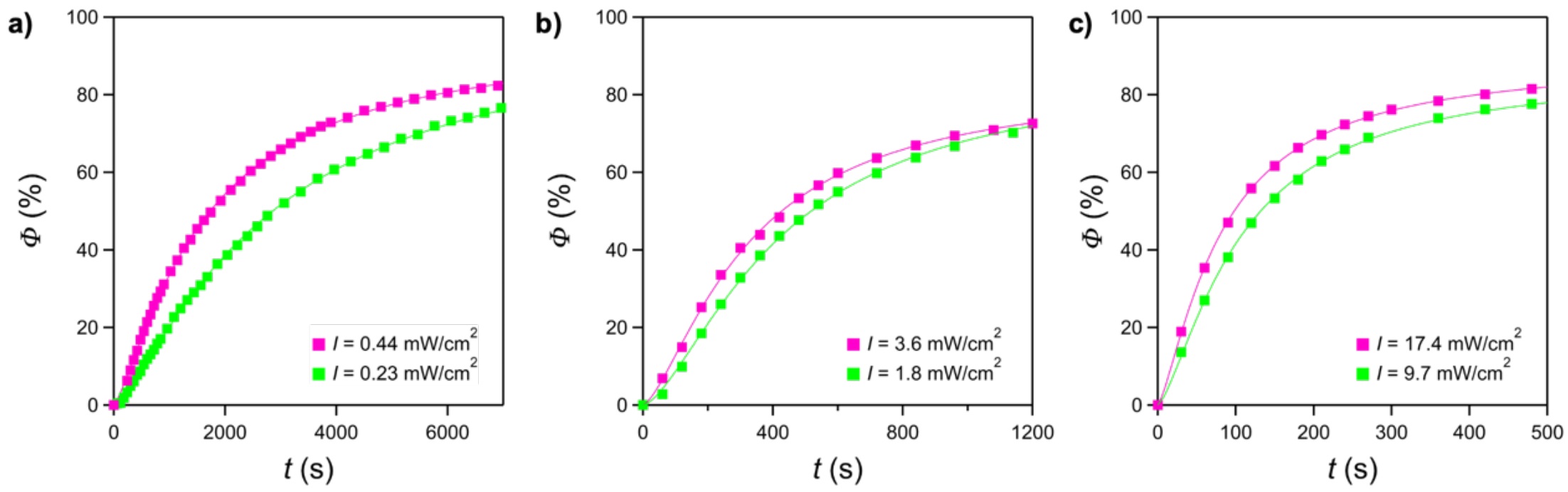

Figure S3. The monomer conversion, Φ, as a function of irradiation time, *t*. (a-c) The panels show Φ in the high- and low-light intensity regions under irradiation with Patterns L, M, and H, respectively.

References

(1) Protesescu, L.; Yakunin, S.; Bodnarchuk, M. I.; Krieg, F.; Caputo, R.; Hendon, C. H.; Yang, R. X.; Walsh, A.; Kovalenko, M. V. Nanocrystals of Cesium Lead Halide Perovskites ($CsPbX_3$, X = Cl, Br, and I): Novel Optoelectronic Materials Showing Bright Emission with Wide Color Gamut. *Nano Lett.* **2015**, *15*, 3692-3696.

(2) Morinaga, M.; Iwaki, T.; Tanaka, H.; Lagzi, I.; Nakanishi, H. Patterning Perovskite Quantum Dots Using Photopolymerization. *ACS Appl. Mater. Interfaces* **2023**, *15*, 17152-17162.

(3) Lv, H. S.; Huang, S. Y.; Zhao, B. X.; Miao, J. Y. A New Rhodamine B-Based Lysosomal Ph Fluorescent Indicator. *Anal. Chim. Acta* **2013**, *788*, 177-182.
